# Setting up a large set of protein-ligand PDB complexes for the development and validation of knowledge-based docking algorithms

**DOI:** 10.1186/1471-2105-8-310

**Published:** 2007-08-25

**Authors:** Luis A Diago, Persy Morell, Longendri Aguilera, Ernesto Moreno

**Affiliations:** 1 Department of Bioengineering, Faculty of Electrical Engineering, Havana Institute of Technology, Havana 19390, Cuba; 2 Faculty of Bioinformatics, University of Information Science, Havana 19370, Cuba; 3 Center of Molecular Immunology, P.O. Box 16040, Havana 11600, Cuba

## Abstract

**Background:**

The number of algorithms available to predict ligand-protein interactions is large and ever-increasing. The number of test cases used to validate these methods is usually small and problem dependent. Recently, several databases have been released for further understanding of protein-ligand interactions, having the Protein Data Bank as backend support. Nevertheless, it appears to be difficult to test docking methods on a large variety of complexes. In this paper we report the development of a new database of protein-ligand complexes tailored for testing of docking algorithms.

**Methods:**

Using a new definition of molecular contact, small ligands contained in the 2005 PDB edition were identified and processed. The database was enriched in molecular properties. In particular, an automated typing of ligand atoms was performed. A filtering procedure was applied to select a non-redundant dataset of complexes. Data mining was performed to obtain information on the frequencies of different types of atomic contacts. Docking simulations were run with the program DOCK.

**Results:**

We compiled a large database of small ligand-protein complexes, enriched with different calculated properties, that currently contains more than 6000 non-redundant structures. As an example to demonstrate the value of the new database, we derived a new set of chemical matching rules to be used in the context of the program DOCK, based on contact frequencies between ligand atoms and points representing the protein surface, and proved their enhanced efficiency with respect to the default set of rules included in that program.

**Conclusion:**

The new database constitutes a valuable resource for the development of knowledge-based docking algorithms and for testing docking programs on large sets of protein-ligand complexes. The new chemical matching rules proposed in this work significantly increase the success rate in DOCKing simulations. The database developed in this work is available at .

## Background

Improving our understanding of protein-ligand interactions at the molecular level plays an important role in the discovery process of new drug candidates. The Protein Data Bank (PDB) [1] is the main source of structural information on protein-ligand complexes. It is constantly being improved through the addition of on-line tools and links to complementary datasets.

Current virtual screening methodologies for in-silico discovery process of new leads rely on databases of chemical complexes with structural information and also chemical, physical and biological properties, when available. Examples of these datasets are the Cambridge Structural Database [2], NCI [3], ZINC [4] and ChemStar [5]. These databases, however, do not provide clues on the ways these molecules may interact with proteins or other macromolecules, which is important for the task of developing knowledge-based docking algorithms.

Several databases containing information on ligand molecules found in the PDB, and on protein-ligand interactions (e.g. PDB-ligand [6], PLD [7], PDBsum [8], Hic-Up [9], Relibase [10,11] and MOAD [12]) have been released. Furthermore, a few databases collecting data on protein binding sites found in the PDB have been recently reported [13-16]. Creative data mining may provide useful knowledge on protein-ligand interactions from these databases, with potential use in the design of docking algorithms or scoring functions. Testing of docking algorithms, on the other hand, requires a particular processing of both the protein and ligand structures, so that the given docking program has all the data it needs (and in the required format) to perform the simulation. The above mentioned databases were not intended for this purpose.

The number of algorithms available to assess and rationalize protein-ligand interactions has increased during the last years [17,18], but the number of test cases used to validate methods keeps being small (from tens to a few hundreds of complexes) and problem dependent [19]. A few datasets have been developed for testing the performance of some of the most popular docking programs. One of them is the GOLD validation test set [20], comprising 100 different complexes extracted from the Protein Data Bank. FlexX-200 [21] is also a dataset consisting of 200 protein-ligand complexes selected and modified by hand from original PDB files. The developers of the DOCK program [22] also compiled a small dataset of 49 small molecules in complex with a variety of macromolecular targets [23]. In these datasets, the structural and chemical information is stored in the right format used by the corresponding programs.

In this work, we report the compilation of a large set of protein-ligand complexes extracted from the Protein Data Bank, which is tailored both for extracting knowledge on these interactions via data mining and for large scale testing of docking algorithms. The term "large scale" means the use of thousands of protein-ligand complexes that were identified in the PDB and processed in an automatic manner. A key element in the extraction procedure was the use of our own definition of molecular contact to identify protein-ligand interactions at the atomic level. The database of protein-ligand complexes was further enriched in molecular properties such as atom types, atomic charges, etc, needed for the scoring functions of many of the docking programs.

In the final part of this paper, we demonstrate how our new database can be a valuable resource in the process of developing knowledge-based docking algorithms. We carried out a research exercise that started with data mining of protein-ligand interatomic interactions, leading then to a modification of the matching rules of the chemical filter used by the program DOCK to speed up the generation of ligand orientations in the binding site, and finished with testing the new rules and comparing them with the old ones by running docking simulations for several hundreds of protein-ligand complexes.

## Methods

### Identifying small ligands in complex with proteins in the PDB

Determining whether a PDB entry contains a non-covalent complex of a protein with one or several small molecules (ligands) may be a complicated task for several reasons. First, there is not explicit information in the PDB entries that would make clear which records contain atomic data belonging to each small molecule, if present in the structure. Usually, small ligands are listed within HETATM records, but this doesn't apply to amino acids or nucleic acids, which may also constitute small ligands. Furthermore, some small molecules are a mixture of amino acids and/or nucleic acids with other chemical groups, being listed both under HETATM and ATOM records. On the other hand, some of the small molecules present in the PDB, especially many sugars, are covalently bound to proteins, being therefore not of interest for the purpose of this work. The solution given here to these problems was to analyse the atomic connectivity within a PDB entry.

We wrote our own program (called 'complex_info') to process the whole PDB. For peptides and nucleotide chains, we set a limit of eight and four residues, respectively, to consider them as ligands. First, all the amino acid and/or nucleotide chains present in an entry were identified. Entries containing nucleotide chains having more than four residues were not further analysed. Water molecules and metal ions were stored apart. Hydrogen atoms, if present, were deleted (and constructed later for ligands, see below).

The remaining heteroatoms, as well as the small peptides and nucleotide chains were all processed together and grouped by their connectivity into fragments (molecules). Two atoms were considered to be covalently bonded if the distance between them was less than 2.1 Å. This number was selected to cover the larger covalent distances we found in the PDB (between sulphur atoms: 2 – 2.05 Å). Fragments having less than 10 atoms were removed, as well as those fragments that were covalently bonded to a protein chain.

Ligands were classified in seven categories according to their chemical nature: peptide, nucleotide, hetero, peptide-nucleotide, peptide-hetero, nucleotide-hetero and peptide-nucleotide-hetero. Ligand residues listed under the ATOM keyword in the PDB files were identified as peptide or nucleotide by checking their residue names. Every residue listed under the HETATM keyword was included in the broad category of 'hetero'.

### Computing protein-ligand contacts

Atomic contacts between two molecules are typically determined based on a distance criterion. Thus, in several reports a contact between two atoms is registered if the distance between them is below a fixed cut-off value. For example, in Relibase [10,11], a value of 7 Å was used. In a previous work, we used a cut-off of 4.3 Å to collect protein-small ligand interactions [24]. In a stricter approach, a contact is postulated if the distance between the two atoms is not larger than the sum of their van der Waals radii, plus certain tolerance value (generally in the range of 0.5 – 1.0 Å) [7,10,11,24].

**Figure 1:**
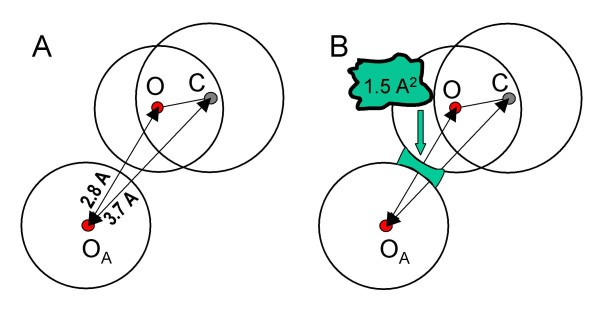
Illustration of the new definition of intermolecular atomic contact. A) Distance criterion: all atoms within a threshold distance are in contact. According to this, the oxygen O_A _and the carbon are considered as being in contact. B) Surface criterion: two atoms are in contact only if their corresponding surfaces are close enough across an area of at least 1.5 Å^2^.

In this work, we introduced a new definition of atomic contact that takes into account a possible screening of the direct interaction between two atoms, produced by a third atom that is covalently bonded to one of them, as illustrated in Figure 1A. The oxygen O_A _and the carbon atom represented in this figure would be considered as being in contact if a purely distance criterion is used. Following our definition, however, they are not contacting each other.

Our definition of atomic contact takes also into account a distance criterion, but with an additional refinement. In our approach, the Connolly's molecular surfaces [25] of the two interacting molecules are employed to determine whether two atoms are in direct contact. The surfaces were calculated using a dot density of 4 Å^-2 ^and a radius of 1.4 Å for the rolling probe. The van der Waals atomic radii were taken (with just a slight rounding) from the AMBER united-atom force field [26].

So, two atoms belonging to different molecules are said to be in contact if: a) the distance between them is not greater than the sum of their van der Waals radii plus a tolerance distance (0.5 Å), and b) if both atoms have associated surface points located around a line joining their centres, and covering at least an area of 1.5 Å^2 ^for each of the two atoms (see Figure 1B).

Following this definition, the program complex_info computes, for each protein-ligand complex, a list of atomic contacts, as well as a more concise list of the protein residues that make contact with the ligand.

### Interface water molecules and ions. Buried surfaces

The program complex_info also determines which of the water molecules, if present in a PDB entry, are located at a protein-ligand interface. In addition, it gives the total surface area of the ligand and the surface areas buried on the ligand and on the protein as a result of the interaction. This analysis was based on the calculated Connolly's surfaces, since his program MS DOT [25] specifies in the output file which of the surface dots (and their associated areas) are buried by other atoms surrounding the molecule of interest.

For each water oxygen that is close to the ligand (distance < 3.5 Å), its Connolly surface was computed taking into account all the surrounding atoms within a cut-off distance of 8 Å. Water molecules having > 90% of their surface buried by ligand and protein atoms were considered as interface water. The same type of analysis was performed for any metal ion present in the structure.

The calculation of the ligand surface buried by the protein was performed including the interface water molecules and ions as if they were part of the protein. Conversely, the interface water and ions were considered as extensions of the ligand surface to compute the area of the protein surface buried by the ligand.

### Typing protein and ligand atoms

When studying molecular interfaces it is important to consider the nature of the atomic interactions, for example, the presence of hydrogen bonds, salt bridges or hydrophobic contacts. For this purpose, it is necessary to know what chemical properties the atoms at the molecular interface have. This information, however, is not included in a PDB file. In particular, the absence of hydrogen atoms in most PDB structures makes it difficult in many cases to determine whether a polar atom can be a donor or an acceptor of a hydrogen bond. In order to overcome these problems, we implemented a procedure for automatic atom type assignment.

For ligand atoms, a few atom types were defined on top of the chemical elements for nitrogen, oxygen and carbon (see Table 1). Nitrogen atoms were classified as donor (N_d), acceptor (N_a), positive (N_p), aromatic (N_r) or "hydroxyl" (N_h, see below); oxygen atoms as acceptor (O_a), negative (O_n) or hydroxyl (O_h); and carbons were divided into aliphatic (C_l) and aromatic (C_r). Similar classifications have been reported elsewhere, for example, the one made by Abola *et al. *[27] for automated analysis of interatomic contacts in proteins.

**Table 1:** Atom types defined in this work and their correspondence with MOL2 types

Atom type	Description	MOL2 types
C_l	Aliphatic carbon – sp3 hybridized	C.1, C.2, C.3
C_r	Aromatic carbon – sp2 hybridized, included in aromatic rings and in guanidine or carboxyl groups	C.ar, C.cat, C.2
N_+	Positive nitrogen – *sp*2 or *sp*3 hybridized, protonated, forming a group with a positive charge (e.g. guanidine group)	N.4, N.pl3
N_d	Nitrogen donor of a hydrogen bond – *sp*2 hybridized, protonated (e.g. amine group)	N.2, N.ar, N.am
N_a	Nitrogen acceptor of hydrogen bond – not all valences covered by heavy atoms, not protonated	N.2, N.ar, N.3, N.1
N_r	Aromatic nitrogen – *sp*2 hybridized, with all its valences covered by heavy atoms	N.ar, N.pl3
N_h	"Hydroxyl" nitrogen in histidines – *sp*2 hybridized, undefined protonation	N.2
O_a	Oxygen acceptor of hydrogen bond – not protonated	O.2, O.3
O_-	Negative oxygen – negatively charged (e.g. nitro group, carboxyl group)	O.co2
O_h	Hydroxyl oxygen – protonated (OH group)	O.2, O.3

Protein atoms were classified according to seven categories: donor, positive, acceptor, negative, hydroxyl, hydrophobic and aromatic, without indicating explicitly the chemical element. This classification matches the definition of chemical types for the "attached point" representation of protein binding sites, as described in [24], designed to be used with the program DOCK. The assignment of atom types for the protein receptors was performed simply from the standard PDB atom names for amino acids. Aspartic and glutamic acids were considered as not protonated, and therefore their carboxylic oxygens were classified as negative. The delta and epsilon nitrogens of histidine were classified as "hydroxyl", as a way to leave undefined their protonation state.

For ligand molecules, atom typing was carried out using the well-known program Babel-1.6 [28], with a few modifications. One of the changes made in the program had the purpose of translating its internal atom types into the newly defined types. Other changes were introduced to correct some errors that we obtained when processing a group of test molecules, as explained below.

Babel-1.6 uses bond distances, bond angles and torsion angles to assign atom types, using methods similar to those described in [29,30]. After determining the chemical element and hybridization corresponding to each atom, hydrogens are placed on atoms to fill up the empty valences. The algorithms implemented in Babel work fine when the geometry of the molecule does not deviate much from the ideal values. The small molecules found in the PDB, however, often show large deviations from optimal bond distances and angles.

To cope as much as possible with these problems, we modified some of the parameters coded in Babel-1.6. Furthermore, we added a criterion based on measuring improper dihedral angles (the dihedral angle determined by an atom and three of its connected atoms) to differentiate between sp2 and sp3 hybridizations. If an atom has three connections to heavy atoms and the absolute improper dihedral is smaller than 18 degrees, then it is sp2 hybridized (18 degrees is halfway between the optimal value for a sp2 atom and that of a sp3 atom [29,30]). New cut-off values for some geometric parameters were obtained by analysing a group of molecules with sulphate, phosphate and carboxyl groups (see Table 2).

**Table 2:** Statistics from the Babel 1.6 runs on the DOCK 4.0 dataset to test the protonation

Chemical element	Number of atoms	Errors *	%
Nitrogen	85	41	48
Oxygen	128	0	0
Carbon	871	1	0.1
Others	30	0	0

Total	1114	42	3.7

*) Number of atoms carrying a different protonation, as compared with the original dataset files.

Errors in the protonation resulted either from an incorrect assignment of the hybridization or from an incorrect filling of a valence of a sp2 nitrogen in a ring structure.

The assignment of 'donor' and 'acceptor' atom types performed by Babel 1.6 for some nitrogen atoms, which depends on their protonation, was not always correct. This kind of problem appeared mainly with heterocyclic structures. To improve the capabilities of Babel to protonate correctly these structures, we included some empirical rules generally accepted in organic chemistry [31].

**Figure 2:**
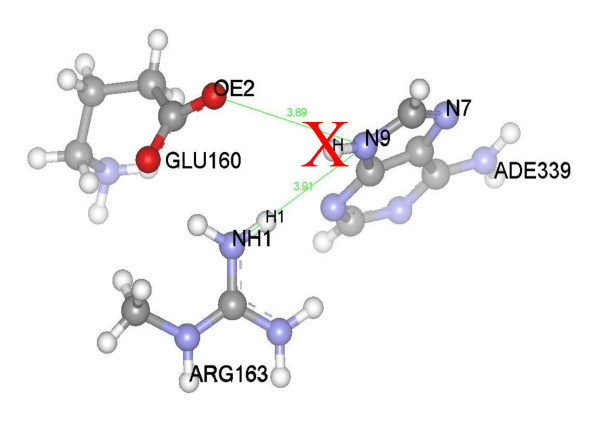
Protonation of the ligand molecule by analysing the protein environment. Protonation of nitrogen atom N9 of molecule ADE339 depends on three parameters: the distance from ligand atom N9 to protein atom NH1 of ARG163, and the angles H-N9-NH1 and H1-NH1-N9. If hydrogen atoms H and H1 fall within a cone described by a solid angle of 60 degrees, then the hydrogen atom attached to the ligand ADE339 is removed (as indicated with a red X), even though the atom OE2 of GLU160 is in a favourable position to accept a hydrogen bond.

Finally, the protonation of nitrogen atoms in ligand ring structures was checked against the crystal structure of the corresponding protein-ligand complex. The influence of every polar protein atom contacting with a ring nitrogen in a ligand was evaluated. This evaluation took into account the distances between the nitrogen and the protein atoms and the angle formed by a possible hydrogen bond donated by the ligand nitrogen. The influence is positive if hydrogen placement favours the formation of a hydrogen bond, but is negative if the added hydrogen is facing a hydrogen from a donor atom in the protein, as illustrated in Figure 2. The final decision on whether to keep or remove the hydrogen atom from the ring nitrogen is taken after weighting all the influences from the neighbouring polar atoms.

Once a molecule is protonated Babel 1.6 can also assign partial charges to atoms, using the Gasteiger algorithm [32]. The information on atom types, hybridization and partial charges was stored in a separate file for each ligand.

### Testing the atom typing with reference datasets

Two reference datasets were used to test the typing of ligand atoms with the modified Babel-1.6 program: the DOCK 4.0 dataset [23], consisting of 49 small molecules randomly selected from the Current Medicinal Chemicals (CMC) molecular database (MDL Information Systems, San Leandro, CA), which was used for testing the implementation of the empirical rules to protonate nitrogen atoms in heterocyclic structures, and the FlexX-200 validation dataset [21], which consists of 200 protein-ligand complexes selected from the PDB. For each complex in the FlexX-200 dataset, an active site file for the proteins as well as two MOL2 files for the ligand (one file with the crystal geometry and another file with a protonated, energy minimized structure) are provided. The FlexX-200 dataset was used to check the performance of the empirical rules introduced in Babel to protonate nitrogen atoms and the final refinement of the protonation against the structures of the protein-ligand complexes. To perform these tests, hydrogen atoms were removed from the original files, which were converted from the MOL2 to the PDB format. The obtained PDB files were then used as input to the Babel program.

### Filtering to create a "non-redundant" dataset

A filtering procedure was applied to the collected protein-ligand complexes aiming to create a non-redundant dataset for data mining. The term "non-redundant", for this particular work, means that the dataset should not contain entries for which both the structures of the ligand and the protein-binding site are similar. Following this principle, the comparison of the complexes was focused mainly on the protein amino acids and the ligand atoms that are in contact with each other. Thus, we compared the ligand structures and the protein-ligand contact tables. The comparison took into account the atom types previously assigned.

First, the filtering procedure was performed within each PDB entry. Afterwards, all pairs of protein-ligand complexes obtained after the first round were compared against each other. If both the ligand structures and the contact tables for two complexes were similar, the complex with lower resolution was discarded.

Comparison of the ligand structures was centred on the atoms contacting the protein. From the list of protein-ligand contacts computed by our contact definition, a graph representation of the interaction is built to find the molecular similarity between two structures. The maximum common sub-graph (MCS) between two structures is used to express molecular similarity. From the MCS, an analysis of the protein-binding site is carried out to eliminate redundancies. Since finding the MCS is a NP-complete graph-matching problem, it is computationally expensive to locate an optimal mapping in a realistic time frame without the application of heuristics. Several approaches have been reported to tackle this kind of problem. Here, our heuristic to find MCS starts with a clique detection algorithm, as described in [33], which constrains the match using our previous atom type definition and successively adds small subgraphs of both ligands. The similarity of the ligands in terms of three-dimensional structure is also taken into account by checking the root mean square deviation (RMSD) of the MCS for every newly included subgraph.

### Data mining study on interactions between atom types

The assignment of atom types carried out in this work, together with all the structural information stored in the database, allows performing different types of studies regarding the chemical nature of the protein-ligand interactions. The extracted knowledge may have important applications, for example, in the design of docking algorithms. In this paper, we explored the data mining possibilities given by our new database of protein-ligand complexes by compiling statistics about the frequencies of interaction between different protein and ligand atom types, and using the obtained data to tune the chemical matching filter implemented in the well known program DOCK [34].

In this study we introduced the term "relative contact frequency" (FCr_ij_) for different pairs of ligand-protein atom types, e.g. N_d-acceptor, which was defined as follows:

FCr_ij _= k Cij/(p_i _p_j_), where Cij is the number of contacts counted for ligand atom type "i" and protein atom type "j", divided by the total number of computed ligand-protein contacts; p_i _and p_j _are the proportions in which atom types i and j, respectively, are found in the database; and k is a normalization constant, computed so that the sum of the frequencies by all protein-ligand pairs yields one.

### Simulations using the program DOCK with a few modifications

To test the performance of different versions of the chemical filter implemented in the program DOCK, we ran a series of simulations with this program for a selected set of hundreds of protein-ligand complexes.

The algorithm implemented in the program DOCK uses a representation of the binding site based on points ("spheres"), which are intended to occupy positions favourable for ligand atoms to be placed there. Then, sets of ligand atoms are matched onto sets of spheres to generate different orientations of the ligand [22]. A chemical filter may be used in this process to speed up the calculations, by discarding matches that do not fulfil a defined set of chemical complementarity, or matching rules [34]. In order to use this chemical filter, chemical labels (colours) must be assigned both to site points and ligand atoms.

Modifying the chemical filter that comes by default with the program DOCK was easily done by just replacing the original input files containing atom type definitions and matching rules.

To create a negative image of the active site, we used our own method for generating binding site points, called "attached points" (ATPTS) [24]. In this approach, the site points are generated in an automated way from templates constructed for each amino acid type, and chemical labels are assigned to these points also in an automatic fashion. Each ATPTS point is linked to a protein atom (its "parent atom") and its label or colour carries chemical information on this atom in its particular chemical context. Furthermore, each ATPTS point is located at a distance from its parent atom which is optimal for an intermolecular atomic interaction.

ATPTS binding site representations were generated in a totally automated way, following the algorithm described in [24]. The selection of the protein residues that form the binding site was made on the basis of their proximity to the ligand in the crystal complex: every protein residue within a distance of 5 Å from at least one of the ligand atoms was included. Bump distances of 2.2 and 2.8 Å were used for polar and nonpolar protein atoms, respectively. A merging distance of 1 Å was used to fuse points of the same colour that were too close to each other.

Since we were interested only in the functioning of the orientation part of the program DOCK with different versions of the chemical filter, we did not perform any scoring of the generated ligand geometries. Instead, we ran "truncated" docking simulations – the code of the program was slightly modified to output to a file the RMSD value, with respect to the crystal structure geometry, of every generated ligand orientation, and then skip the scoring routines. The docking simulations were carried out also in automated way, using simple scripts.

## Results and discussion

We processed the whole PDB release of August 2005 (32069 files) using our program 'complex_info' in order to detect ligand molecules bound to proteins, extract a variety of information on these protein-ligand complexes and calculate several atomic and molecular properties. As result, 22513 complexes from 10694 files were extracted and analysed.

### Typing of ligand atoms

One important part of the calculations performed on the extracted protein-ligand complexes was the assignment of atom types to both the protein and the ligand. We defined a simple set of atom types, as described in the Methods section (Table 1), aiming mainly at classifying the molecular interactions in a few elementary types such as hydrophobic contacts and hydrogen bonding. We decided to employ the popular program Babel-1.6 to perform the typing of ligand atoms. One important reason behind this decision was the possibility of modifying the code to implement our new atom type definitions, and making other changes as necessary.

Typing of protein atoms was easily done from the PDB atom names. Typing of ligand atoms, however, presented some difficulties due mainly to the imperfect stereo chemistry of ligand molecules extracted from the PDB and imprecisions in the protonation of nitrogen atoms.

**Figure 3:**
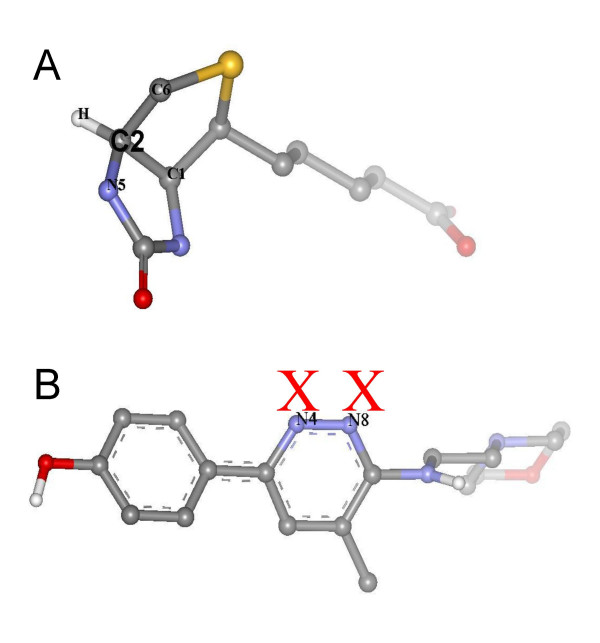
Examples of molecules that were correctly protonated by the modified Babel program. A) Biotin. The atom C2 was correctly typed as sp3, and then a hydrogen was added to fill the empty valence; B) Moxiraprine. The hydrogen atoms marked with red X's were removed by applying an empirical rule that allows correct protonation of this type of ring.

First we tested the performance of the original Babel-1.6 in protonating correctly polar atoms and assigning correct atom types on the DOCK 4.0 dataset. As result, 32 out of 49 molecules were reported as having errors related mainly to nitrogen atoms in heterocyclic structures. All these errors were eliminated after introducing the modifications that were explained in the Methods section. For example, in the Biotin molecule (see Figure 3A) the atom C2, which is *sp3*-hybridized, has an average bond angle higher than the cut-off value set in the Babel-1.6 code for this type of hybridization. For this reason, it was assigned an incorrect *sp2 *hybridization. By checking the improper dihedral angle formed by C2 and its connected atoms C1, C6 and N5, a correct *sp3 *hybridization was assigned. For the Moxiraprine molecule (see Figure 3B), a *sp2 *hybridization was correctly assigned by Babel to atoms N4 and N8, but the protonation of these atoms was wrong because Babel tries to fill up all empty valences with hydrogens. By adding an empirical rule stating that a nitrogen in a six-member ring with all its atoms *sp2*-hybridized is not protonated, the problem was solved. Table 2 shows some statistics from the test runs on the DOCK 4.0 dataset.

In a second step, the modified Babel-1.6 was tested on the FlexX-200 dataset, using the ligand structures with their geometries extracted directly from the PDB. Some of these molecules had atoms that in the original PDB files are covalently bonded to the protein, and consequently had a modified protonation. These atoms were not taken into account. Table 3 shows the obtained results.

**Table 3:** Errors in atom typing/protonation process with FlexX-200 dataset, for the original and modified versions of the Babel 1.6 program

Chemical element	Number of atoms	Babel (original)		Babel (modified)	
		Errors	%	Errors	%
Nitrogen	431	54	12.5	34	7.8
Oxygen	1036	4	0.3	0	0
Carbon	2979	40	1.3	0	0
Others	120	1	0.8	0	0

Total	4566	99	2.2	34	0.7

The final checking of the protonation performed against the crystal structures of the protein-ligand complexes allowed to correct some of the remaining errors in the protonation of ligand nitrogen atoms, but only for those atoms in contact with the protein receptors. Therefore, some errors still remained for nitrogen atoms that are outside the protein-ligand contact tables. Similar problems have been reported for other atom-typing programs [35]).

### Selecting a non-redundant set of protein-ligand complexes

Once the atom typing was performed for all the extracted complexes, a filtering procedure was carried out with the purpose of creating a non-redundant dataset, following the principles delineated in the Methods section. First, obvious redundancies such as repeated complexes at different resolutions and repeated copies of the same ligand within an entry were eliminated. In most cases, these copies are found for proteins having several identical binding sites, for instance, the cholera toxin B pentamer (entry 3chb), or for entries containing copies of the same complex in the asymmetric unit of the crystal. In either case, the protein-ligand contact patterns displayed by the different ligand copies are almost identical.

On the other hand, there are several small groups of entries containing the same protein, but in complex with different ligands, or mutants of the same protein in complex with the same or different ligands. We did not eliminate any of these complexes a priori because each of them might contribute different protein-ligand contacts. For instance, different versions of the same protein very often contain mutations in the binding site region, which most likely results in distinct new contacts with the ligand.

After a first filtering round where copies of the same ligand within the same PDB entry were removed, 12360 complexes out of 22513 remained. Afterward, a second filtering round was carried out where all complexes were compared against each other. As result, 6586 complexes were obtained.

### Some statistics extracted from the collected protein-ligand complexes

**Figure 4:**
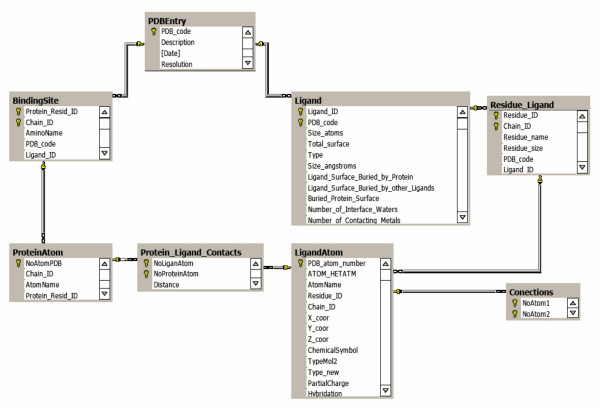
Implementation of the database in a SQL Server.

**Figure 5:**
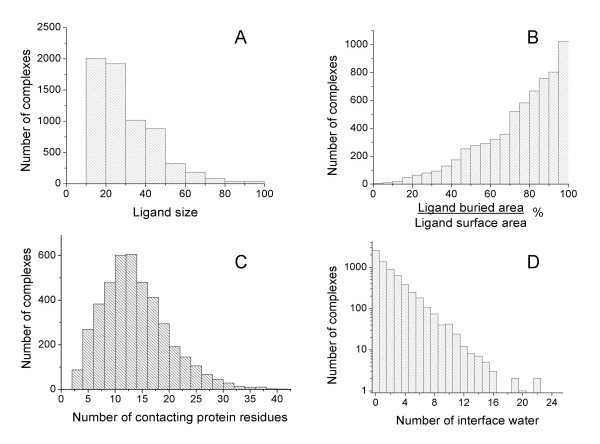
Examples of statistical data extracted from the database. A) Distribution of ligand size; B) Percent of ligand surface buried by the protein; C) Number of protein residues per complex that contact the ligand molecule; D) Number of water molecules at the protein-ligand interface.

**Figure 6:**
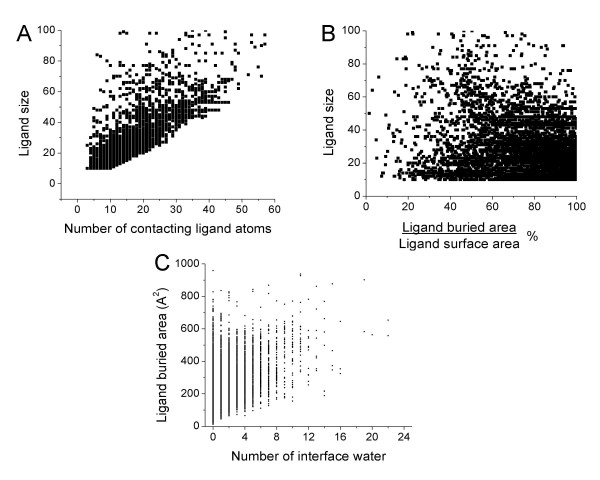
Relationships between different variables in the database. A) Number of ligand atoms contacting the protein vs. the ligand size; B) Percent of ligand surface buried by the protein vs. the ligand size; C) Number of water molecules at the protein-ligand interface vs. the ligand surface buried by the protein.

All information about extracted protein-ligand complexes was stored in a SQL server database, as illustrated in Figure 4. The implementation of the database in a SQL server makes it easy to perform different types of queries. Figures 5 and 6 show, as examples, some collected statistics from the database, using histograms and correlation plots. These statistics were collected for the selected non-redundant set. Figure 5A shows the distribution of ligand sizes expressed as the number of heavy atoms. As the number of complexes where the ligand has more than 100 atoms is very small, only ligands smaller than 100 atoms are shown in the histograms.

As shown in Figure 5B, in most of the complexes of the database the ligand has more than 60% of its surface in contact (buried) with the protein. Fifteen protein residues as average are contacting ligand atoms (Figure 5C), with a contact area smaller than 1000 Å^2 ^for all complexes. The buried ligand surface is always slightly smaller than the corresponding buried protein surface because the ligand is embedded within the protein site and because of the presence, in many cases, of water molecules at the interface. Figure 5D shows the important role that water molecules play in many cases, serving as bridges in protein-ligand interactions. About 5500 complexes out from the selected 6586 contain interface waters, with several hundreds of these complexes containing five or more. There are a few complexes with more than 20 water molecules at the protein-ligand interface.

Some of these properties are correlated, for example, Figure 6A shows that the higher the ligand size the higher the number of ligand atoms making contact with the protein. However, there is no correlation between the fraction of ligand surface buried by the protein and the ligand size, as shown in Figure 6B. Most of the smallest ligands are almost or completely embedded in the protein, but there are also many of them that make contact with only a fraction of their surfaces. For larger ligands (> 50 heavy atoms), in most cases a big part of the molecule is outside the binding site. Figure 6C shows that there is no correlation between the number of water molecules at the protein-ligand interface and the buried ligand area.

Also by doing simple queries to the database, it is possible to know how the different atom types defined in this work are represented in the PDB ligands, and their ratio of participation in contacts with the protein receptors. Table 4 shows these results for the selected non-redundant set. Nitrogen and oxygen atoms in ligand molecules have a higher ratio of contacts with proteins than carbon atoms, although the *sp2*-hybridized "aromatic nitrogens" (N_r) have a low contact ratio, due to the fact that their three valences are occupied by heavy atoms that screen most part of their atomic surface. Negatively charged oxygens have the highest ratio of interaction (~75%).

**Table 4:** Number of ligand atoms and number of contacts with protein amino acids by ligand atom type, extracted from the non-redundant set selected from the database

Atom Type	Number of atoms (Na)	Contacts (C)	C/Na
C_l	51223	23891	0.466
C_r	25287	12000	0.475
N_+	1633	1061	0.650
N_d	6257	4031	0.644
N_a	4722	3090	0.654
N_r	2448	426	0.174
O_a	15286	8272	0.541
O_-	12503	9277	0.742
O_h	9939	6893	0.694
Others *	4928	-	-

Total ^†^	129298	68941	0.533

*) Atoms corresponding to chemical elements other than C, O or N.

†) The total values do not include the unclassified atoms ("others")

### Data mining in the database: Application in docking

We further performed a series of data mining and docking simulations, in order to show the potentialities of our database in terms of extracting useful data that could be incorporated into knowledge-based docking algorithms, and testing these algorithms using a large number of protein-ligand complexes. For this purpose we used two different non-redundant data sets compiled from the database. One of them, called PDB2003 and containing 4243 complexes, was obtained from the complexes deposited in the PDB before March 2003, using the filtering procedure explained in the Methods section, while the second dataset, called PDB2005 and having 2931 complexes, was obtained from the rest of the structures, up to August 2005. PDB2003 was used to derive statistics on protein-ligand interactions by atom type, whereas PDB2005 was employed in testing newly designed chemical matching rules, as described below.

### Protein-ligand interactions by atom type

We further queried the database to determine the number of interatomic contacts by both ligand and protein atom types. Table 5 collects the results from these queries. As shown in this table, hydrophobic contacts between carbon atoms account for most of the collected interactions, while some strong interactions such as those between a positively charged nitrogen and a negatively charged oxygen are less represented. These results, obviously, are a direct consequence of the large differences among atom type populations in the database.

**Table 5:** Number of contacts in the database between the defined ligand atom types and protein atom types, as extracted from the PDB2003 dataset

Ligand atom type	Number of contacts	Number of contacts with protein atom types *						
		
		ACC	NEG	DON	POS	HDX	PHO	ARM
C_l	11404	600	694	507	473	634	3145	5351
C_r	5900	304	79	171	168	228	1884	3066

N_+	569	80	263	15	19	57	37	98
N_d	1784	533	406	71	52	159	173	390
N_a	1407	33	23	169	165	150	393	474
N_r	231	43	0	1	2	7	53	125

O_a	3750	229	110	567	754	389	703	998
O_-	5147	307	181	818	2009	1313	216	303
O_h	3863	404	1333	473	565	380	275	433

Total	34055	2533	3089	2792	4207	3317	6879	11238

*) Protein atoms types are: acceptor (ACC), negative (NEG), donor (DON), positive (POS), hydroxyl (HDX), hydrophobic (PHO) and aromatic (ARM).

We then defined what we called "relative contact frequencies" (FCr) for ligand-protein atom type pairs, aiming at removing the biases in contact frequencies caused by the differences in atom type populations. The relative contact frequencies were obtained by normalising the values displayed in Table 5 according to the total number of contacts and the population frequencies of the different ligand and protein atom types, as explained in the Methods section. The results of these calculations are shown in Table 6.

**Table 6:** Probabilities of interaction between the ligand atom types and protein atom types

Ligand atom types	Protein atom types						
	
	ACC	NEG	DON	POS	HDX	ALP	ARM
C_l	0.0104	0.0099	0.0080	0.0050	0.0084	0.0201	0.0210
C_r	0.0102	0.0022	0.0052	0.0034	0.0059	0.0233	0.0232

N_+	0.0279	0.0752	0.0047	0.0040	0.0152	0.0048	0.0077
N_d	0.0593	0.0370	0.0072	0.0035	0.0135	0.0071	0.0098
N_a	0.0047	0.0027	0.0216	0.0140	0.0162	0.0204	0.0151
N_r	0.0369	0.0000	0.0008	0.0010	0.0046	0.0168	0.0242

O_a	0.0121	0.0048	0.0272	0.0240	0.0157	0.0137	0.0119
O_-	0.0118	0.0057	0.0286	0.0466	0.086	0.0031	0.0026
O_h	0.0207	0.0561	0.0220	0.0175	0.0149	0.0052	0.0050

After the performed normalization, it turned out that atomic interaction pairs formed by a positively charged nitrogen in the ligand and a negatively charged atom in the protein (an oxygen atom from the carboxyl group of asparagine or glutamine) have the highest relative frequency in protein-ligand complexes. In general, positively charged and hydrogen bond donor nitrogen atoms in ligands have high relative frequencies of interaction with hydrogen bond acceptors and negatively charged atoms in the protein. On the other hand, negatively charged oxygens in ligands interact with high relative frequencies with positively charged nitrogens and hydroxyl oxygens. Ligand hydroxyl oxygens are more prompt to donate a hydrogen bond that accepting one from the protein.

In summary, interactions between charged and polar atoms have much higher relative frequencies than interactions with or between hydrophobic or aromatic atoms. This means that when present both in the ligand molecule and in the protein binding site, nitrogens and oxygens have a much higher probability of being involved in interactions with the protein than individual carbon atoms.

To reflect these relative contact preferences by atom type in a discrete way, the interactions were classified into three categories: highly probable, probable and less probable, as defined in Table 7. It should be noted that the probability intervals were chosen ad-hoc, aiming to include only a very few types of interaction in the "highly probable" and "probable" categories.

**Table 7:** Classification of the interactions based on interaction probabilities

Type	Classification	Probability interval
0	Less probable	≤0.0216
1	Probable	0.0216–0.0360
2	Highly probable	≥0.0360

### Evaluating chemical complementarity in protein-ligand complexes

With the aim of making a practical use of the obtained probabilities of interaction by atom types, we designed a set of chemical complementarity rules that could be used as a filter to discard wrong ligand orientations by a docking program, such as DOCK [34].

As explained in Methods, DOCK uses a representation of the binding site based on points. Then sets of ligand atoms are matched onto sets of points to generate different orientations of the ligand. A chemical filter implemented in the program helps discarding matches that do not fulfil a defined set of chemical (or matching) rules, thus speeding up the calculations.

Here we used our own method to generate binding site points, called "attached points" (ATPTS), as briefly explained in Methods and in more details in ref. [24]. Each ATPTS point carries a label that reflects the chemical nature of its parent protein atom. Not as a casual coincidence, these labels correspond to the atom types that were defined in this work for protein atoms. Since a spatial matching of an ATPTS point with a ligand atom corresponds to a contacting interaction between its parent atom and this ligand atom, we can extrapolate the statistics obtained for ligand-protein contacts to ligand-ATPTS matches.

Thus, ATPTS binding site representations were generated for the 4243 protein-ligand complexes included in the PDB2003 dataset, in a totally automated way. The calculation time per structure was, on average, less than 0.1 s.

The quality of the ATPTS representation of protein binding sites, in terms of its success in matching point and ligand atom positions for the correct (crystallographic) orientation of ligand molecules, was thoroughly demonstrated in a previous paper [24]. For this purpose, we defined and calculated a magnitude called "number of matching atom-point pairs" (N_mp_). A matching atom-point pair is formed by a site point and a ligand atom being within certain "matching cut-off distance" (1.5 Å) from each other. Then we showed that for the vast majority of the tested complexes, more than 70% of the ligand atoms that are in contact with the protein are matched by ATPTS points. Here we repeated the same type of calculation for the PDB2003 dataset and obtained roughly the same result (not shown).

**Figure 7:**
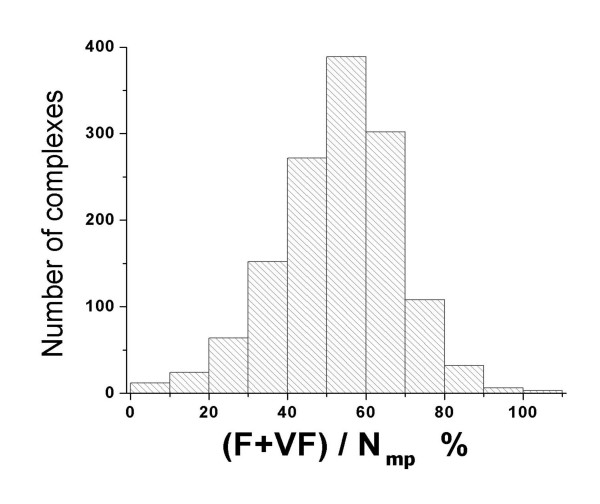
Distribution of probable (F) and very probable (VF) protein-ligand complexes, given in percent relative to the number of matching pairs. A cut-off matching distance of 1.5 Å was used to compute the ligand atom-attached point matching pairs.

In this work, taking profit of the atom type data included in the database, we could also evaluate the quality of the formed matching pairs, according to the classification of the atomic interactions defined in Table 8. The results of this evaluation are shown in Figure 7. For most of the complexes in the PDB2003 dataset, more than 40% of the matching pairs correspond to probable (F) or very probable (VF) interactions.

**Table 8:** Classification of the different types of protein-ligand atom contacts in three categories: less probable (0), probable (1) and very probable (2)

Ligand atom types	Protein atom types						
	
	ACC	NEG	DON	POS	HDX	ALP	ARM
C_l	0	0	0	0	0	0	1
C_r	0	0	0	0	0	1	1

N_+	1	2	0	0	0	0	0
N_d	2	2	0	0	1	0	0
N_a	0	0	1	0	0	0	0
N_r	2	0	0	0	0	0	1

O_a	0	0	1	1	0	0	0
O_-	0	0	1	2	2	0	0
O_h	0	2	1	0	0	0	0

### Defining and testing new matching rules for DOCKing simulations

If the number of matching pairs with probable and very probable interactions is high enough, the stringency of the matching rules to be used in the docking runs may be increased, for example, by enabling only matching of very probable pairs in the ligand orientation process. This would significantly accelerate the calculations by reducing the number of ligand orientations to generate and evaluate. On the other hand, the use of these rules must allow the generation of a sufficient number of correct orientations, otherwise the program may fail in predicting a right solution.

Here we defined two different sets of matching rules: one that permits very probable and probable interactions (R1), and a second set that permits only very probable matches (R2). Then we tested their performance as compared with the chemical complementarity rules coming with the DOCK program (R_DOCK_), which are much more permissive.

The test of the three sets of matching rules was carried out for a selected set of complexes from the PDB2005 dataset. To rule out other variables that may affect the results of a docking simulation, such as big ligand size and a small contact area with the protein, which are not the subject of this particular study, we restricted the test set to complexes in which ligands have between twenty and forty heavy atoms and more than 70% of their molecular surface embedded in the protein binding site. As we were interested in evaluating the accuracy of the postulated matching rules, the chosen complexes also should have atom types included in the rules sets R1 and R2. Finally, 542 complexes were selected from the PDB2005 dataset, and ATPTS binding site representations were generated for all of them.

For all the selected complexes we ran "truncated" docking simulations, as explained in Methods. No scoring was carried out since we were not interested in the performance of the scoring functions, but only in evaluating the number of correct ligand orientations that are produced by the program. The maximum number of orientations to be generated was limited to 10^5^. This number was high enough to explore all the combinations generated by the matching algorithm for most of the complexes, and was much higher than the value used by default (10^3^). Ligands were kept rigid in these simulations.

In order to check the efficiency of the chemical rules, we computed the number of "good" ligand-receptor configurations generated by the DOCK program in the ligand orientation process. As "good orientations" were considered those whose RMSD value with respect to the crystal geometry was less than 2 Å.

**Figure 8:**
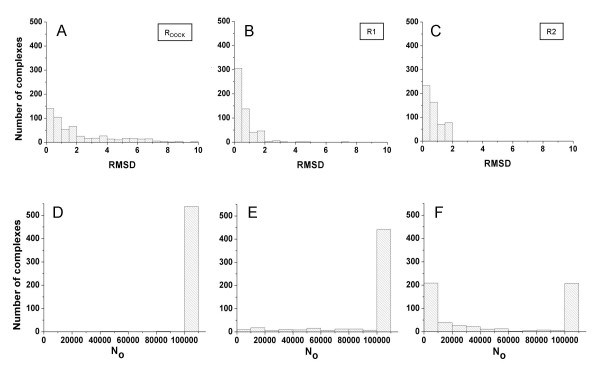
Results from the docking simulations performed for 542 complexes from the PDB2005 dataset, using three different sets of chemical matching rules. Panels A, B and C show the distribution of RMSD values of the best found solution using the native DOCK, R1 and R2 sets of matching rules, respectively. Panels D, E and F show the distribution of the total number of orientations (N_0_) explored in the simulations, for the same sets of matching rules.

The simulations for the 542 complexes took just a few hours. A comparison of the results obtained for the three sets of matching rules is shown in Figure 8. The comparison is focused on the analysis of the RMSD of the best orientation generated by the DOCK chemical matching algorithm, the total number of orientations explored (N_0_) and the number of "good" orientations obtained.

When using its default set of chemical matching rules, the program DOCK fails in producing a good solution for about 30% of the complexes (Figure 8A), even though the program reached, for almost all the complexes, the maximum number of ligand orientations that was allowed (Figure 8D). For the 70% of complexes showing a successful result, an average number of 2985 good solutions was produced by the program.

Using the R1 set of matching rules significantly improves the performance of the simulations, with only a very few complexes having their best orientation with an RMSD > 2 Å (Figure 8B). For this set of rules the number of possible ligand atom-site point matches was still high enough as to make the program produce the allowed 10^5 ^orientations for most of the complexes (Figure 8E). The average number of produced good solutions was 4062.

Finally, when the R2 rules were employed, a good solution was always found (Figure 8C). For this more restrictive set of rules, the number of explored orientations was reduced notably, as shown in Figure 8F, and nevertheless, a high number of good orientations was obtained (2138 as average) for each protein-ligand complex. These results demonstrate that the matching rules used to limit the exploration of the conformational space between the protein and the ligand can be more stringent and still produce very good results if the ligand molecules have atom types with statistically high probabilities of interaction with proteins.

On the other hand, the fact that using the matching rules given by default for the DOCK runs made the program fail in about 30% of the tested cases causes concern. Since the ensemble of ligand atom-site point matches produced with the R1 or R2 rules must be a subset of the ensemble of matches produced using more permissive rules, or not using any filter at all, the reason for the failure must be that the maximum allowed number of ligand orientations was not enough to give the program the chance of exploring the right sector of the orientational space. Even for small and medium size ligands, the total number of atom-point combinations that can be produced may be so large, that the probability of obtaining correct matches within the first 10^5^, or even more unfavourably, within the first 10^3 ^explored conformations, can be small.

## Conclusion

We compiled a large database of small ligand-protein complexes derived from the PDB that currently contains more than 6000 non-redundant structures. In processing the PDB structures, we characterized in details the protein-ligand interactions, using a new definition based on the proximity of the molecular surfaces. Furthermore, the structural data was enriched with chemical properties that we calculated using our own as well as existing algorithms. As an example to demonstrate the value of the new database for data mining and testing of docking algorithms, we derived a new set of chemical matching rules to be used in the context of the program DOCK, based on contact frequencies between ligand atoms and points representing the protein surface, and proved their enhanced efficiency with respect to the default set of rules included in the program. One important finding in this study is the high rate of failure of the program DOCK in finding orientations close to the crystal structure when using the default chemical filter, even in the case when the maximum number of allowed ligand orientations is set 100 times larger than the default value.

## Authors' contributions

LAD worked in the typing of ligand atoms, the filtering of the data to create non-redundant sets, the data mining to extract several statistics from the database and in the design and testing of the new rules for chemical matching. He also was in charge of most of the manuscript writing. LA designed and leaded the work to structure the data into a SQL server. PM designed and built a user-friendly graphical interface with wide possibilities for querying the database on a web server. EM conceived, designed and coordinated the project, created the program to identify and extract structural information on the protein-ligand complexes, and took an active part in manuscript writing. The four authors read and approved the final version of the manuscript.
